# The Impact of Instagram on Dental Professionals in Saudi Arabia: A Cross-Sectional Investigation

**DOI:** 10.3290/j.ohpd.c_2231

**Published:** 2025-08-26

**Authors:** Khalifa S. Al-Khalifa, Fadak H. Almarar, Fatimah M. Alatiyyah, Fatimah A. Alhassan, Raghad T. AlJarboua, Yasmin I. Alhamdan, Esraa M. Alabdurubalnabi

**Affiliations:** a Khalifa S. Al-Khalifa Associate Professor of Dental Public Health, Department of Preventive Dental Sciences, College of Dentistry, Imam Abdulrahman Bin Faisal University, Dammam 31441, Saudi Arabia. Writing – review and editing, writing – original draft, visualisation, supervision, project administration, methodology, investigation, formal analysis, conceptualisation.; b Fadak H. Almarar General Dentist, Dental House Clinic, Khobar 34414, Saudi Arabia. Writing – review and editing, writing – original draft, data curation, methodology, and investigation.; c Fatimah M. Alatiyyah Teaching Assistant, Department of Genetic Research, Institute for Research and Medical Consultations (IRMC), Imam Abdulrahman Bin Faisal University, Dammam 31441, Saudi Arabia. Writing – review and editing, writing – original draft, data curation, methodology, and investigation.; d Fatimah A. Alhassan Resident, Fellowship in Periodontics Programme, College of Dentistry, Umm Al-Qura University, P.O. Box 715, Makkah Al-Mukarramah 21955, Saudi Arabia. Writing – review & editing, writing – original draft, and data curation.; e Raghad T. AlJarboua Resident, Fellowship in Orthodontics Programme, College of Dentistry, Imam Abdulrahman Bin Faisal University, P.O. Box 1982, Dammam 31441, Saudi Arabia. Writing – review and editing, writing – original draft, data curation.; f Yasmin I. Alhamdan Resident, Fellowship in Prosthodontics Programme, College of Dentistry, Imam Abdulrahman Bin Faisal University, P.O. Box 1982, Dammam 31441, Saudi Arabia. Writing – review and editing, writing – original draft, data curation.; g Esraa M. Alabdurubalnabi Resident, Fellowship in Periodontics Programme, College of Dentistry, Imam Abdulrahman Bin Faisal University, P.O. Box 1982, Dammam 31441, Saudi Arabia. Writing – review and editing, writing – original draft, data curation.

**Keywords:** Instagram, social media, dentist, patient engagement, dental practices, Saudi Arabia, cross-sectional study

## Abstract

**Purpose:**

This study aimed to assess the utilisation of Instagram primarily as a marketing tool among dentists in Saudi Arabia and its perceived impact on patient engagement.

**Materials and Methods:**

A cross-sectional survey was conducted among 385 dentists using a convenience sampling method. The questionnaire collected data on demographics, Instagram usage patterns, perception of marketing strategies on the platform, and factors influencing the selection of a dentist or dental clinic. Data were analysed using IBM SPSS Statistics and presented as descriptive statistics and bivariate analyses.

**Results:**

A total of 385 responses were received, yielding a response rate of 64.2%. Approximately 77.1% of participants reported regular Instagram use, with nearly half accessing the platform more than three times per day. Most respondents indicated using Instagram for personal purposes (42.9%) and marketing (39%). The most effective marketing strategies identified were paid promotional advertisements (75%), Instagram searches (55%), and patient recommendations (50.1%). Key content-related factors enhancing account appeal included clinical case photos (84.9%) and high-quality images (99%). Dentists working in the private sector were more likely to utilise Instagram for marketing and reported a significant increase in patient flow (60%) as a result.

**Conclusion:**

Instagram serves as a valuable marketing platform for Saudi dental professionals, particularly in the private sector. The platform enhances patient outreach, practice visibility, and brand building. Further research is recommended to explore ethical guidelines, content strategies, and potential applications in professional and patient education.

The rapid growth of social media usage has transformed communication, information exchange, and marketing worldwide. Globally, social media usage has increased by 9.2%, with Saudi Arabia experiencing an 8.7% rise.^4^ Notably, Saudi Arabia has one of the highest social media penetration rates per capita, with approximately 27 million active users— 76.4% of whom use Instagram.^28^

Instagram is a highly visual and interactive social media platform that enables users to share images, videos, and private messages. Its features support self-expression, documentation, and real-time engagement, which contribute to its widespread popularity across various age groups.^4,21,23^ As a platform unconstrained by geography or time, Instagram has become an essential tool for businesses and professionals to connect with audiences, including in the healthcare sector.^7^

For dental professionals, Instagram offers unique advantages for visual communication, patient education, and practice marketing. Dentists can use the platform to showcase treatment outcomes, share oral health information, and build relationships with current and potential patients.^3,12,26^ A study in Saudi Arabia found that 66.6% of dental patients follow dentists on Instagram,^4^ while in the United States, over half of dental professionals use social media – including Instagram – for marketing purposes.^16^ Other studies have highlighted Instagram’s role in promoting dental residency programs^12^ and enhancing reputation management within orthodontic practices.^19^

Despite its visual strengths and algorithm-driven visibility, Instagram’s full potential for dental marketing remains underutilised, particularly in Saudi Arabia. Existing literature lacks sufficient exploration of how dental professionals in the region use Instagram and how they perceive its impact on patient communication and business growth.^5,12^ This gap highlights the need for research focused on professional use and the effectiveness of Instagram in the dental field.

Therefore, the aim of this study was to assess the utilisation of Instagram primarily as a marketing tool among dentists in Saudi Arabia and its perceived impact on patient engagement.

## Research Objectives

To examine how Saudi dentists use Instagram for marketing.
To identify Instagram features perceived as effective for patient engagement.

### MATERIALS AND METHODS

#### Study Design, Participants, and Settings

A descriptive, cross-sectional survey was conducted among dentists practising in Saudi Arabia. The minimum required sample size was calculated using G*Power based on an assumed response distribution of 50%, a 95% confidence level, a 5% margin of error, and an estimated population of 19,622 dentists according to the Ministry of Health Statistical Yearbook 2020.^13,18^ These parameters yielded a minimum sample size of 377 participants. A convenience sampling technique was used. Eligible participants included general dentists, specialists, and consultants from both the public and private sectors. Dentists of all genders were invited to participate, while non-Saudi dentists or those practising outside Saudi Arabia were excluded to maintain a culturally and professionally homogeneous sample, as non-Saudi dentists may differ in their practice rights, patient communication styles, and marketing autonomy.

#### Ethical Considerations

After the institutional review board approval of Imam Abdulrahman bin Faisal University was obtained, the questionnaire was distributed (IRB-2022-02-222, dated 6 May 2022). Consent was obtained from the participants who agreed to participate in the study. The consent contained information regarding the type of the study, objectives, procedures, beneﬁts, and duration of answering the questionnaire. Confidentiality and privacy of the participants’ responses were assured.

#### Measurement of Study Variables

A self-administered survey was conveniently distributed among dental practitioners who were asked through different social media platforms such as WhatsApp, Instagram, Facebook and LinkedIn to complete the survey. The questionnaire was adapted from previously validated surveys developed by Al-Khalifa^4^ and Parmar et al,^22^ both of which examined social media use in dental contexts. To ensure content validity in the adapted version, the questionnaire was reviewed by a panel of three experts in dental public health and digital marketing. The panel evaluated each item for clarity, relevance, and cultural appropriateness to the Saudi dental community. Minor modifications were made based on their feedback.

Pilot testing was conducted with 20 dentists to assess question clarity and user acceptability. Based on their input, three refinements were made: (1) two questions were reworded for clarity; (2) two redundant items on Instagram tools were merged; and (3) Likert-scale labels were standardised across all items.

Hard and soft copies of the questionnaire were distributed based on each dentist’s preference. The survey was composed of 17 questions divided into four parts. The first part included 6 questions on demographic information. The second section comprised 4 questions related to Instagram usage among dentists, including the types of social media accounts used, the purpose of using social media, frequency and timing of account checks, whether patients follow their Instagram accounts, and whether they communicate or interact with patients through Instagram. The third section covered 2 questions on marketing perception strategies among dentists, which explored dentists’ views on effective Instagram marketing tools. Participants rated various strategies using a consistent 3-point scale: ‘very important’, ‘important’, or ‘not important’. The fourth section (3 questions) assessed dentists’ different Instagram engagement strategies with patients. It included preferred communication method (Direct messages, Instagram stories or Instagram posts), frequency of posting interactive Instagram stories (Yes/No), and whether consultations are provided via Instagram (Yes/No). The last section (2 questions) assessed the factors affecting the selection of a dentist/ dental clinic from the point of view of dentists, using the same 3-point importance scale.

#### Statistical Analysis

All the data collected from the dental practitioners were transferred to an EXCEL sheet for statistical analysis. Descriptive statistics of frequency distribution and percentages were calculated for the categorical variables. Chi-square tests were performed to determine the relationship between the use of different social media among dental practitioners. For statistical purposes, a P value <0.05 was considered statistically significant. All statistical analysis was performed using SPSS version 22.

### RESULTS

A total of 385 responses were received out of 600 invitations, yielding a response rate of 64.2%. Table 1 represents the demographic data of participants. A majority of the participants (75.8%) were between 24 and 34 years of age, and 65.7% were males. About half the participants were specialists/consultants (52.5%) and worked in the private sector (48.3%). The highest participation in the study was from the Eastern Province (35.1%), followed by the Central region (26.2%) and the Western region (20%). More than 50% of the participants reported an income level below 20,000 SAR ($5,333) monthly.

**Table 1:** Demographic data of study participants (n = 385)

Variables	N (%)
**Age**	
24–34 years	292 (75.8)
35 and above years	93 (24.2)
**Gender**
Male	253 (65.7)
Female	132 (34.3)
**Specialty**
General practitioner	183 (47.5)
Specialist/consultant	202 (52.5)
**Practice area**
Government	159 (41.3)
Private	186 (48.3)
Unemployed	40 (10.4)
**Region**
Eastern	135 (35.1)
Western	77 (20.0)
Central	101 (26.2)
Northen	18 (4.7)
Southern	54 (14.0)
**Monthly income**
<20,000 SAR	226 (58.7)
>20,000 SAR	159 (41.3)


A majority of the participants (77.1%) reported that they use Instagram regularly compared to other social media platforms. About 40% of the participants use Instagram for personal purposes (42.9%) and check their Instagram accounts more than three times daily (46.2%). The participants tend to navigate Instagram mostly in the evenings (69.1%) and have been using social media accounts for more than 6 years (61.8%). About one-third of the participating dentists (33.2%) believe that their patients are following their Instagram accounts, and two-thirds (66.8%) answer inquiries asked virtually. When the participating dentists were asked how their patients discovered their Instagram account, their response was through Instagram search (32.5%) and recommendation from an Instagram user (21.6%) (Table 2).

**Table 2:** Instagram use among study participants (n = 385)

Variables	N (%)	Variables	N (%)
**Type of SM used regularly**	**Are patients following the Instagram account?**
Instagram	297 (77.1)	Yes	128 (33.2)
Twitter	42 (10.9)	No	56 (14.5)
Facebook	4 (1.0)	Sometimes	82 (21.3)
Snapchat	30 (7.8)	I don’t know	119 (30.9)
TikTok	12 (3.1)		
**Main purpose of using Instagram**	**Response to questions**
Personal	165 (42.9)	Yes	257 (66.8)
Educational	70 (18.2)	No	25 (6.5)
Marketing	150 (39.0)	Sometimes, not applicable (I have not received questions)	103 (26.8)
**How often to check Instagram?**	**How did patients find the Instagram account?**
Once weekly	23 (6.0)	Paid promotion ad	35 (9.1)
Once daily	73 (19)	Account is advertised by the clinic	42 (10.9)
2–3 times a day	111 (28.8)	Through a recommendation of an Instagram user (mention sticker)	83 (21.6)
More than 3 times a day	178 (46.2)	Through Instagram search	125 (32.5)
**Time of the day to use Instagram**	Through hashtags	12 (3.1)
Morning (8 am–12 pm)	28 (7.3)	I don’t know	88 (22.9)
Afternoon (12 pm–5 pm)	91 (23.6)	
Evening (After 5 pm)	266 (69.1)
**How long of using SM account?**
1–3 years	81 (21.0)
4–6 years	66 (17.1)
More than 6 years	238 (61.8)


**Fig 1:**
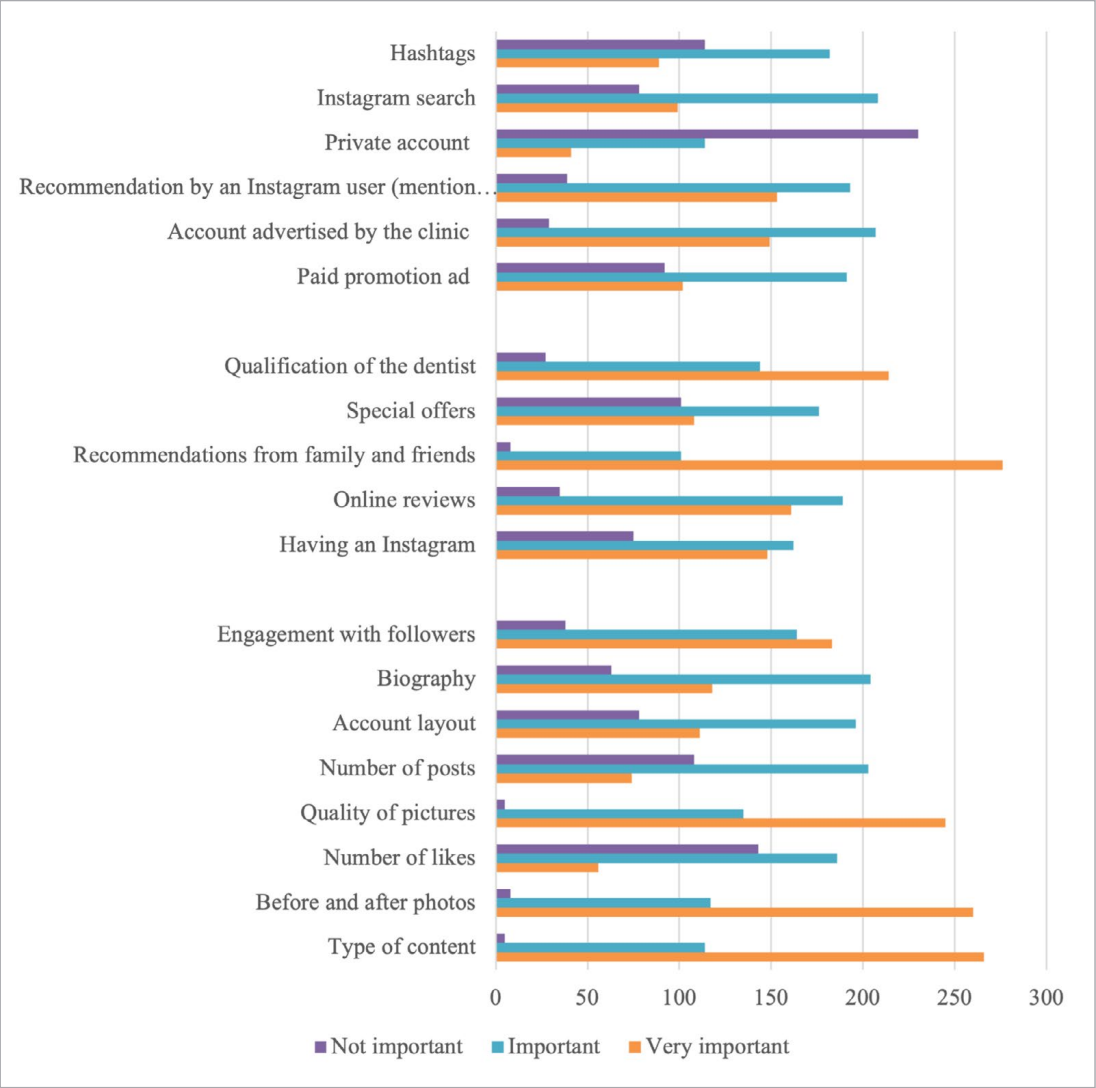
Participants’ perception of dental marketing strategies and factors that affect the patients’ choice of dentists and attractiveness of Instagram accounts (n = 385).

As for the most effective strategies to promote a dental practice on Instagram, about 3 out of 4 participants believe it was very important/important to use paid promotion advertisements. More than half of the participants (53.8%) agree it was important to advertise for their account by the clinic and through a recommendation by another Instagram user. Regarding the use of a private account to promote practice, more than half of the participants (59.7%) responded that it was not important, and 55.0% agreed that an Instagram search of the dental practitioner was important. In this study, 47.3% of the participants thought it was important to use hashtags (#) to promote their practice. Regarding the factors affecting the patient’s choice of dentists, about half of the participants agreed that it was important/ very important to have an Instagram account (80.5%) as well as having online reviews (90.9%). The majority of participants (97.9%) believed that recommendations from family and friends, as well as the qualification of the dentists (93%), were important/very important when choosing a dentist. In terms of choosing dentists who provide special offers, it was seen as an important/very important factor by more than half of the participants (73.8%), as seen in Figure 1.

Referring to the factors that enhance the attractiveness of an Instagram account, most of the participants (98.7%) considered that the type of content was an important/very important factor that contributes to its attractiveness. Nearly (98%) of the participants responded that before-and-after photos, number of likes (63%), pictures’ quality (99%), and number of posts (72%) were important/very important factors to attract patients on Instagram. About 80% of participants thought that the account layout and biography (84%) were important/very important in the account attractiveness. With respect to followers’ engagement, 90.1% of participants admitted it was an important/very important factor in patients’ attractiveness to their Instagram accounts (Fig 1).

Half of the participants agreed that the most successful tool for Instagram engagement was through direct messages (49.9%). A majority reported they posted Instagram stories (71.7%). More than a third of the participants (39.7%) never consulted with their followers. Participants thought that Instagram was the most effective platform for attracting patients (74.0%), and 43.4% agreed it was effective in increasing the flow of patients (Table 3).

**Table 3:** Participants’ responses about successful tools of engagement in Instagram (n = 385)

Variables	N (%)
**Method of communicating with patients**
Direct messages (DM)	192 (49.9)
Instagram stories	57 (14.8)
Instagram posts	39 (10.1)
I don’t usually communicate with my patients through Instagram	97 (25.2)
**Posting Instagram stories**	
Yes	276 (71.7)
No	109 (28.3)
**Giving consultation to patients**
Always	47 (12.2)
Sometimes	185 (48.1)
Never	153 (39.7)
**Most effective platform in attracting patients**	
Instagram	285 (74.0)
Twitter	31 (8.1)
Facebook	2 (0.5)
Snapchat	31 (8.1)
TikTok	36 (9.4)
**Flow of patients increased**	
Yes	167 (43.4)
No	28 (7.3)
I don’t know	190 (49.4)


Table 4 represents the participants’ responses to the most attractive content to share. The majority of the participants (84.9%) agreed that sharing pictures of their own cases was the most attractive content to share. On the other hand, 83.1% thought that pictures from other resources might not be that attractive. Only 39.5% of the participants explained new products and procedures on their Instagram accounts, while 42.1% explained the procedural steps. As for involvement in the community service events, only one-quarter of the participants found it attractive.

**Table 4:** Participants’ response about the attractiveness of the shared content (n = 385)

Variables	Yes N (%)	No N (%)
Pictures of your own cases	327 (84.9)	58 (15.1)
Pictures from other resources	65 (16.9)	320 (83.1)
Explaining new products and procedures	152 (39.5)	233 (60.5)
Explaining the procedures steps	162 (42.1)	223 (57.9)
Involvement in community service events	100 (26.0)	285 (74.0)


Table 5 represents a comparison between different practice areas and different reasons for using Instagram on its utilisation, where the results showed a significant difference between dentists who use Instagram for marketing purposes and in the private sector (P <0.001). Private practice used Instagram for marketing purposes, which had a higher rate (51%) than the unemployed and government; this was highly significant (P <0.001). Interestingly, 60% of those using Instagram for marketing purposes experienced an increase in patient flow (P <0.001). In addition, 48.7% of the dentists who use Instagram for marketing purposes noticed patients are following their Instagram account (P <0.001).

**Table 5:** Comparison between different practice areas, and different reasons for using Instagram on its utilisation (n = 385)

Variables	Practice area	P value	Variables	Main purpose of using Instagram	P value
Government	Private	Unemployed	Personal	Educational	Marketing
n (%)	n (%)
Type of SM used regularly (Instagram)	111 (69.8)	158 (84.9)	28 (70.0)	0.009	Giving consultations to patients (Sometimes)	69 (41.8)	30 (42.9)	86 (57.3)	<0.001
Main purpose of using Instagram (Marketing)	43 (27.0)	96 (51.6)	11 (27.5)	<0.001	Flow of patients increased (Yes)	48 (29.1)	29 (41.4)	90 (60.0)	<0.001
Response to questions (Yes)	88 (55.3)	150 (80.6)	19 (47.5)	<0.001	Patients following the Instagram account (Yes)	58 (35.1)	38 (54.3)	114 (76.0)	<0.001
Method of communicating with patient (Direct messages (DM))	70 (44.0)	108 (58.1)	14 (35.0)	0.001					
Posting Instagram stories (Yes)	101 (63.5)	149 (80.1)	26 (65.0)	0.002					
Patients following the Instagram account (Yes)	61 (38.4)	136 (73.1)	13 (32.5)	<0.001					


### DISCUSSION

This study highlights the widespread adoption of Instagram among Saudi dentists, confirming the platform’s growing relevance in dental practice. Most participants reported frequent Instagram usage, suggesting its deep integration into both their personal routines and professional activities. This consistent engagement reflects broader global trends where social media is becoming a dominant tool for communication and marketing in healthcare.

The findings suggest that Instagram serves multiple functions for dentists, ranging from personal use to strategic marketing. Many participants leverage the platform to enhance professional visibility, showcase clinical work, and interact with current or potential patients. These behaviours align with previous studies that have identified social media, particularly Instagram, as a powerful medium for branding and patient engagement in dentistry.^3,4,27^

This dual usage of Instagram for personal enjoyment and professional marketing reflects a broader trend in the healthcare sector. While the dual use of Instagram for personal enjoyment and professional marketing is increasingly common, it also raises important ethical and legal considerations. Blending personal and clinical content may blur boundaries between professional identity and private life, potentially undermining public trust or exposing practitioners to reputational risks. Additionally, posting patient-related content, even with consent, requires strict adherence to confidentiality and privacy regulations. Dentists must ensure that their social media practices comply with ethical guidelines and legal standards established by regulatory bodies. Developing clear policies on digital professionalism may help clinicians navigate the complexities of maintaining a balanced and responsible online presence.

Demographic patterns reveal that younger professionals, especially those aged 24–34, are leading the use of Instagram in dental settings. This aligns with global social media trends and underscores the comfort younger generations have with digital platforms.^1,29^ Additionally, the high representation of private-sector dentists among Instagram users points to a competitive drive where professionals seek to differentiate their services and attract patients online.^25,29^

Patient engagement through Instagram also appears to be gaining momentum. A significant proportion of dentists reported that their patients follow them on Instagram and interact via messages or inquiries. These interactions illustrate the platform’s value as a communication tool and its potential to strengthen patient relationships. The importance of discoverability – through search functions and referrals – highlights how digital visibility can influence patient behaviour and clinic selection.^8,27^ As such, these findings not only reflect the current landscape of dental practice in Saudi Arabia but also emphasise the evolving nature of patient–dentist interactions in the digital age.

Participants identified several effective marketing strategies, such as paid advertisements and user recommendations, as important for enhancing practice visibility. This suggests that dentists are aware of and actively employing multifaceted marketing approaches that combine both direct promotions and organic outreach. These insights reflect a shift in how dental professionals view social media, not merely as a passive communication channel but as an active component of their marketing strategy.^4,14^

When examining preferences around content visibility, most dentists favoured public profiles and emphasised features that improve discoverability, such as hashtags and profile layout. The value placed on online reviews, personal recommendations, and dentist qualifications also emphasises the multi-dimensional factors influencing patient decisions. These findings suggest that dentists recognise the importance of reputation management and accessibility in the digital landscape.^10,15,22^

Visual content plays a key role in Instagram marketing. High-quality images and original clinical cases were viewed as especially attractive, supporting prior findings that authenticity and aesthetics enhance engagement.^9^ This is particularly pertinent in the dental field, where visual content such as before-and-after photos is compelling and serves as a powerful tool for demonstrating treatment outcomes and building trust with potential patients.^24^ Additionally, the layout of the account and the effectiveness of the biography were deemed important by the majority of participants. A well-organised Instagram profile with a clear and engaging biography can facilitate easier navigation and provide potential patients with essential information about the dental practice, thereby enhancing overall user experience.^31^ Engagement with followers emerged as a crucial factor, with a significant percentage of participants recognising its importance in attracting patients. This finding is consistent with the literature that emphasises the role of active engagement – such as responding to comments and direct messages – in fostering a sense of community and trust between dental practitioners and their patients.^11^ By actively engaging with their audience, dentists can not only promote their services but also create a supportive environment that encourages patient loyalty and referrals.

Interactive features like direct messaging and Instagram stories were reported as useful tools for maintaining audience engagement. These strategies align with the growing body of evidence supporting the effectiveness of real-time, personal communication in building patient trust and loyalty.^8^ Although many dentists do not currently provide consultations through Instagram, this may represent an area for future growth, particularly in the context of expanding telehealth services.^6^ The perception that Instagram is the most effective platform for attracting patients further underscores its importance in modern dental marketing strategies. This is corroborated by studies that indicate social media, particularly Instagram, is increasingly recognised as a vital tool for patient acquisition and retention in dental practices.^27^ Moreover, a majority of participants agreed that Instagram effectively increases patient flow, supporting the notion that a well-managed social media presence can translate into tangible business outcomes. This finding aligns with the broader trend in healthcare marketing, where social media platforms are leveraged to enhance visibility and foster patient relationships.^30^ Overall, these results highlight the importance of strategic engagement on Instagram, emphasising the need for dental professionals to adopt innovative approaches that leverage the platform’s unique features to enhance patient interaction and practice growth.

A noticeable majority of participants indicated that sharing pictures of their own clinical cases was the most attractive content to post. This preference is in agreement with existing literature that emphasises the effectiveness of personal and authentic content in fostering trust and engagement among followers.^9^ By showcasing their own work, dentists not only demonstrate their expertise but also create a more relatable and personal connection with their audience, which is crucial in the healthcare sector where trust is paramount.^9^ On the other hand, a significant portion of participants expressed that pictures sourced from other resources were perceived as less attractive. This finding suggests that original content may resonate more with audiences, reinforcing the idea that authenticity is a key driver of engagement on social media platforms like Instagram.^11^ Furthermore, participants reported explaining new products and procedures on their accounts, including detailed procedural steps. This indicates a potential opportunity for dentists to enhance their educational outreach through social media by providing informative content that can help clarify dental procedures for patients.^17^

The findings of this study have several implications for both clinical practice and policy. As social media, particularly Instagram, becomes an integral part of professional branding and patient interaction, dental practitioners must be equipped with the skills to manage their digital presence effectively and ethically. This calls for the integration of social media marketing and digital professionalism into dental education and continuing professional development programmes. Additionally, there is a pressing need for national regulatory bodies and professional associations in Saudi Arabia to establish clear guidelines for dentists’ use of social media. These guidelines should address issues such as patient confidentiality, informed consent for content sharing, appropriate content boundaries, and the distinction between personal and professional profiles. Such measures will help ensure that digital engagement enhances, rather than compromises, the quality and integrity of dental care.

While this study provides valuable insights into the use of Instagram among Saudi dentists, it is important to acknowledge its limitations. One limitation of this study is that the survey tool has not been fully validated. Some of the questions use response types that make methods like Cronbach’s alpha less suitable. In future research, it would be helpful to use other ways to check reliability, such as test-retest methods or item response theory. The study relies on self-reported data, which may be subject to biases and inaccuracies, including social desirability bias. In addition, recruiting participants via social media introduces selection bias, as it may exclude dentists not active on these platforms. Furthermore, this study included only Saudi national dentists, excluding non-Saudi practitioners. Although all licensed dentists in Saudi Arabia operate under the same regulatory framework, this exclusion may limit the generalisability of the findings to the broader dental workforce in the country. Future research could incorporate quantitative analysis of Instagram data to validate self-reported information. The study’s cross-sectional nature prevents the establishment of causal relationships between Instagram use and professional outcomes. Also, the study did not analyse the quality or impact of specific content types shared by dentists on Instagram, which could provide deeper insights into effective strategies. Lastly, the rapid evolution of social media platforms, including Instagram, may necessitate continuous updates to research methodologies and findings.

Based on the findings of this study, some recommendations can be proposed. Such as conducting longitudinal studies to explore how Instagram use influences long-term patient engagement and practice growth. Also, there can be an investigation into the effectiveness of specific content types, such as educational posts, clinical case showcases, and promotional ads, in attracting and retaining patients. Last but not least, to develop and promote ethical guidelines for using social media in dental practice to ensure professionalism and patient confidentiality.

### CONCLUSION

This study highlights the prominent role of Instagram in modern dental practice among Saudi dentists, particularly younger practitioners and those in private clinics. Instagram is not only a marketing platform but also a means of patient engagement and brand building. The findings underscore the importance of high-quality, personalised content, visual aesthetics, and active interaction in enhancing online visibility and trust.

For dental professionals, these insights suggest the need to adopt thoughtful, ethically sound social media strategies that align with patient expectations and professional standards. For policymakers and regulatory bodies, the results point to the need for clear social media guidelines and training programmes to support responsible digital engagement in dentistry.

By recognising Instagram as a key communication and marketing tool, stakeholders in the dental sector can better support its integration into practice while safeguarding professionalism and patient trust.

#### Acknowledgements

##### Ethical approval and consent to participate

Ethical clearance (IRB-2022-02-222 dated 5-6-2022) was obtained from the Institutional Review Board (NCBE Registration No. HAP-05-D-003) at Imam Abdulrahman bin Faisal University. All participants gave informed written consent before participating in this study.

### Dentist Survey

#### Demographics

##### Age

24 years
25–34 years
35–44 years
45–54 years
>55 years

##### Gender

Male
Female

##### Specialty

General practitioner
Periodontist
Prosthodontist
Restorative dentist
Endodontist
Maxillofacial surgeon
Pediatric dentist
Orthodontist
AEGD
Dental public health

##### Main practice area

Government
Private
Unemployed

##### Region

Eastern region
Western region
Central region
Northern region
Southern region

##### Monthly income

>4000 SAR
5000–10,000 SAR
11,000–20,000 SAR
>20,000 SAR

#### Use of Instagram among dentists

##### How often are you using Instagram?

Once weekly
Once daily
2–3 times a day
More than 3 times a day

##### What time of the day do you use Instagram?

Morning (8 am–12 pm)

Afternoon (12 pm–5 pm)

Evening (After 5 pm)

##### Are your patients following your Instagram account?

Yes
No
I don’t know

##### Do you communicate / respond to questions asked by your patients on Instagram?

Yes
No

#### Perception of dental marketing strategies among dentists

##### How did your patient find your professional / dental clinic’s Instagram account?

Paid promotion ad
Account is advertised by the clinic
through a recommendation of an Instagram user (mention sticker)
through Instagram search
through hashtags
I don’t know

##### Strategies you find most effective in promoting your dental practice

**Table:** 

Statement	Very important	Important	Not important
Paid promotion ad			
Account advertised by the clinic			
Recommendation by an Instagram user (mention sticker)			
Instagram search			
Hashtags			

#### Successful tools of engagement in Instagram

##### What is your method of choice when it comes to communicating with your patients through Instagram?

Direct messages (DM)
Instagram stories (poll stickers, question stickers, emoji slider stickers)
Instagram posts
I don’t usually communicate with my patients through Instagram

##### Do you usually post interactive Instagram stories in your professional/dental clinic Instagram account?

Yes
No

##### Do you give consultations to your patients via Instagram?

Yes
No

#### Factors affecting the selection of a dentist

##### In your opinion, what are the most important factors when choosing a dentist / dental practice?

**Table:** 

Statement	Very important	Important	Not important
Instagram presence			
Online reviews			
Recommendations from family and friends			
Special offers			
Qualification of the dentist			

##### What makes a professional / dental clinic account attractive?

**Table:** 

Statement	Very important	Important	Not important
Original interesting content			
Before-and-after photos			
Number of likes			
Quality of posts			
Number of posts			
Account layout			
Biography			
Engagement with followers			
